# Multiparametric ultrasound imaging for the assessment of normal versus steatotic livers

**DOI:** 10.1038/s41598-021-82153-z

**Published:** 2021-01-29

**Authors:** Lokesh Basavarajappa, Jihye Baek, Shreya Reddy, Jane Song, Haowei Tai, Girdhari Rijal, Kevin J. Parker, Kenneth Hoyt

**Affiliations:** 1 Department of Bioengineering, University of Texas at Dallas, BSB 13.929, 800 W Campbell Rd, Richardson, TX 75080 USA; 2 Department of Electrical and Computer Engineering, University of Rochester, Rochester, NY USA; 3 Department of Electrical and Computer Engineering, University of Texas at Dallas, Richardson, TX USA; 4 Department of Medical Laboratory Sciences, Tarleton State University, Forth Worth, TX USA; 5 Department of Radiology, University of Texas Southwestern Medical Center, Dallas, TX USA

**Keywords:** Kidney diseases, Biomedical engineering

## Abstract

Liver disease is increasing in prevalence across the globe. We present here a multiparametric ultrasound (mpUS) imaging approach for assessing nonalcoholic fatty liver disease (NALFD). This study was performed using rats (*N* = 21) that were fed either a control or methionine and choline deficient (MCD) diet. A mpUS imaging approach that includes H-scan ultrasound (US), shear wave elastography, and contrast-enhanced US measurements were then performed at 0 (baseline), 2, and 6 weeks. Thereafter, animals were euthanized and livers excised for histological processing. A support vector machine (SVM) was used to find a decision plane that classifies normal and fatty liver conditions. In vivo mpUS results from control and MCD diet fed animals reveal that all mpUS measures were different at week 6 (*P* < 0.05). Principal component analysis (PCA) showed that the H-scan US data contributed the highest percentage to the classification among the mpUS measurements. The SVM resulted in 100% accuracy for classification of normal and high fat livers and 92% accuracy for classification of normal, low fat, and high fat livers. Histology findings found considerable steatosis in the MCD diet fed animals. This study suggests that mpUS examinations have the potential to provide a comprehensive estimation of the main components of early stage NAFLD.

## Introduction

**Figure 1:**
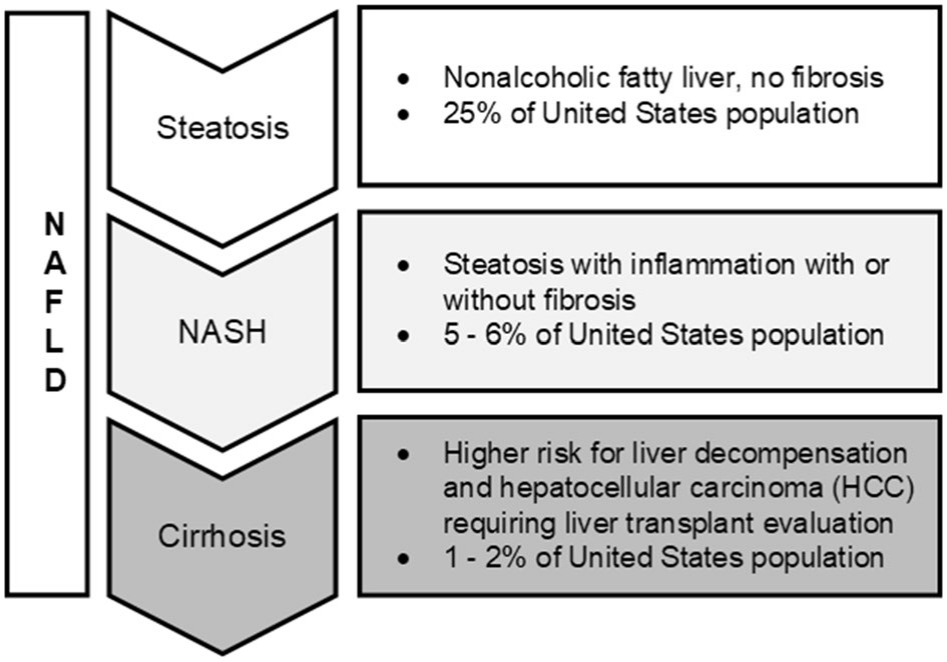
Spectrum of nonalcoholic fatty liver disease (NAFLD) ranging from simple steatosis through stages of hepatocyte injury (inflammation, nonalcoholic steatohepatitis or NASH), and eventually to end stage liver cirrhosis and increase likelihood of hepatocellular carcinoma (HCC) development.

Nonalcoholic fatty liver disease (NAFLD) is one of the comorbid conditions associated with the worldwide obesity epidemic and affects between 80 to 100 million individuals in the United States alone^1,2^. This disease is an increasingly prevalent clinical syndrome associated with obesity and type 2 diabetes mellitus^3^. Most cases of NAFLD are asymptomatic and diagnosis of this disease only often comes to light due to testing for other liver-related issues. As summarized in Fig. 1, NAFLD is the aggregate term defining the disease state that includes steatosis, nonalcoholic steatohepatitis (NASH), and cirrhosis.

Individuals that develop simple steatosis alone are generally thought to have a relatively benign long-term prognosis. However, up to a quarter of these patients may develop NASH, which can then progress to late-stage scarring (cirrhosis)^4^. This latter condition is known to be a potent risk factor for the development of hepatocellular carcinoma (HCC) in susceptible individuals. Given the current prevalence of NAFLD, it is anticipated that NASH-induced cirrhosis will become the most common symptom for liver transplantation in the future^5^. The ability to differentiate simple steatosis from NASH is critically important for the clinical management of NAFLD patients. Evidence to date confirms that early stage NASH may have a 40% probability of progressing to more advanced stages of hepatic fibrosis within 8 years^6–8^. Given the prevalence of NAFLD, it has even been suggested that early liver biopsy might be needed in all NAFLD patients as it is expected that earlier intervention and more aggressive treatment may reduce overall mortality^9^.

Conventionally, a diagnosis of NASH requires an assessment of liver histology. Liver biopsy is therefore considered the reference standard to detect and stage liver cell injury from NASH^10^. However, liver biopsy has several disadvantages, including sample error, interrater and intrarater variability, poor patient acceptance (especially on patient follow-up), and potential complications such as bleeding and death^11–13^. Serologic tests have high accuracy for differentiating advanced fibrosis from mild or no fibrosis^14–16^, but they are poor for diagnosing mild fibrosis and necroinflammation. Consequently, tremendous effort has also been devoted to the development of imaging techniques that can noninvasively detect, accurately stage, and reliably monitor NAFLD.

Contrast-enhanced ultrasound (CEUS) uses microbubble (MB) contrast agents that are confined to the vascular space to improve visualization of blood flow and the measurement of tissue perfusion kinetics^17^. It is now known that during the early stages of NAFLD development, fat-laden hepatocytes become swollen, and in NASH, further swelling occurs due to hydropic change (ballooning) of hepatocytes leading to sinusoidal distortion. Consequently, both intrasinusoidal volume and microvascular blood flow are reduced up to 50% of control^18,19^. This observation was supported by a recent 2019 study that also demonstrated CEUS imaging was more sensitive in diagnosing early stage fatty infiltration-mediated microvascular changes in liver parenchyma and provided a NASH diagnosis in mouse models with greater accuracy^20^. If unhindered, hepatic vein transit time and intrahepatic circulatory time progressively decrease with liver disease severity due in part to arteriovenous shunting and a hyperdynamic circulatory state (i.e., temporal kinetic changes in MB contrast agent flow)^21,22^. In the case of NASH, this progressive blood flow derangement within the liver precedes liver fibrosis development^23^ and any of the eventual complications of cirrhosis, such as HCC and portal vein thrombus^22^. It is worth noting that fibrotic changes also increase the absolute peak of contrast enhancement during CEUS imaging since arteriovenous shunts reduce the rate of MB dissolution as the contrast agent bypasses capillary beds^24^.

The use of noninvasive ultrasound (US) for tissue characterization has been the focus of research efforts for several decades now, where the challenge is to find hidden patterns in the US data to reveal more information about tissue function and pathology that cannot be seen in conventional US images. H-scan US is a new imaging approach that closely links the mathematics of Gaussian-weighted Hermite (*GH*) functions to the physics of scattering and reflection from different tissue structures^25–29^. Specific integer orders, termed *GH*_*n*_, are related to the *n*th derivative of a Hermite function. After attenuation correction^30^, matched filters employing specific orders of *GH*_*n*_ functions can be used to quickly analyze the spectral content of backscattered US signals and to colorize the display, providing pixel-level discrimination between different-sized US scatterers. In general, low frequency spectral content is generated from larger scattering structures whereas high frequency signal content is produced by an US wave interacting with smaller scatterer aggregates of scale below the wavelength of the US transmit pulse. We envision that progressive formation of diffuse fat vacuoles (macrovesicular steatosis) throughout early NAFLD progression will alter both the B-scan and H-scan US signal and reflect liver pathology.

Given the spectrum of NAFLD ranges from simple steatosis, through stages of liver cell injury (steatohepatitis), to fibrosis, and eventually to cirrhosis, it is appropriate to ask whether biomechanical imaging of the liver has a complementary role in NAFLD management. Shear wave elastography (SWE) is a noninvasive technique that uses both US and low-frequency elastic waves to quantify select biomechanical parameters related to the tissue environment. In short, SWE uses an US pulse to create shear waves that travel perpendicular to, and much slower than the longitudinal US waves, making it possible to accurately track and measure them within a limited distance^31^. From the spatial analysis of shear wave propagation patterns, shear wave speed and attenuation parameters can be estimated. These tissue biomechanical properties have been shown to be intimately related to fat during the development of steatosis^32,33^ and collagen content with increased fibrosis^34,35^.

While SWE measurements can be difficult to obtain in overweight and obese subjects, coupling this data with complementary US-based image findings to form a composite metric (i.e., virtual NAFLD activity score) may help increase the clinical value. Towards this, we introduce a new multiparametric US (mpUS) imaging approach and image-derived biomarkers of liver tissue using an animal model of NAFLD. This method combines CEUS to quantify liver tissue perfusion, SWE to measure viscoelasticity, and H-scan US imaging to estimate US scatterer size and organization. The mpUS imaging was performed at 0 (baseline), 2, and 6 weeks using Sprague–Dawley rats that were fed either a control or a methionine and choline deficient (MCD) diet. These mpUS measurements were used as feature inputs for a support vector machine (SVM) and principal component analysis (PCA) to find a decision plane that classifies normal versus steatotic liver. Histology imaging was performed from the excised liver tissue after 6 weeks of experimentation.

## Results

**Figure 2:**
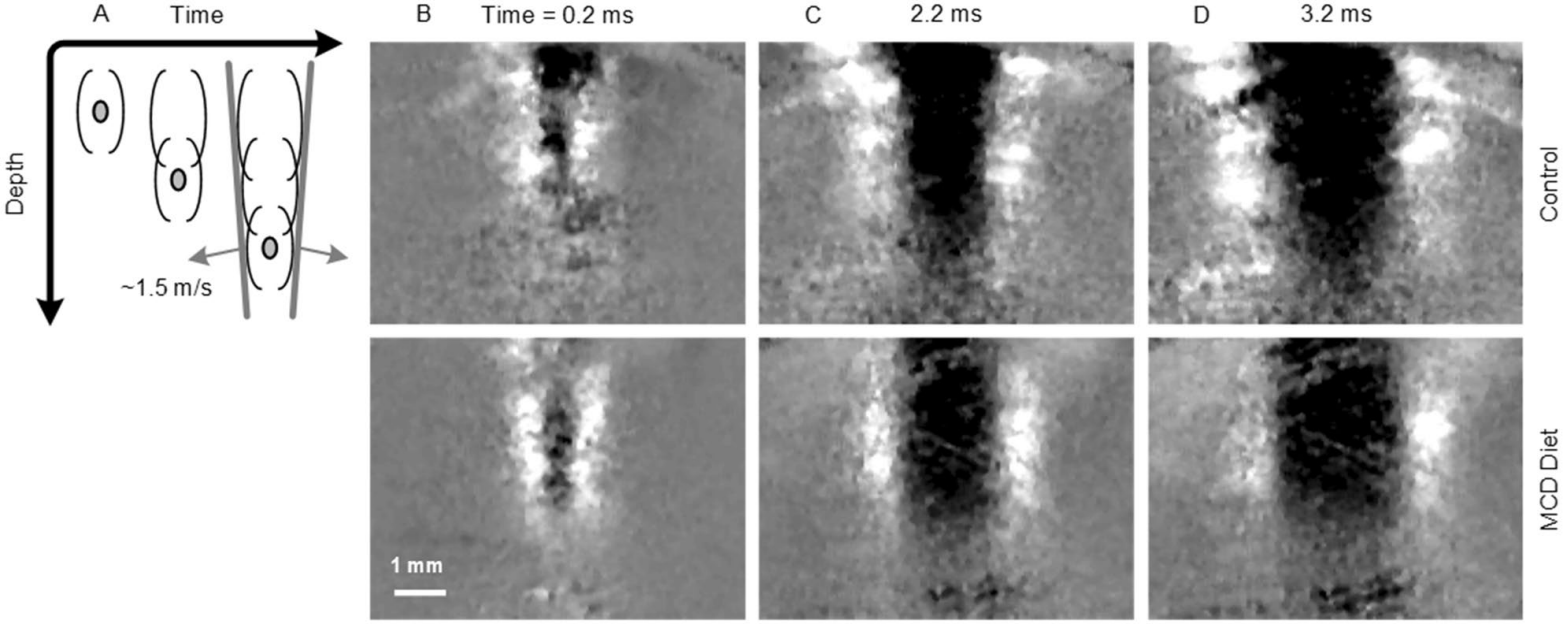
(**A**) Principle of shear wave elastography (SWE) and in vivo ultrasound (US) images detailing shear wave propagation in liver parenchyma from animals fed either a standard chow (control) or a methionine and choline deficient (MCD) diet for 6 weeks. After shear wave excitation (0 ms), the matched series of US images depict differential progression and attenuation at times of (**B**) 0.2, (**C**) 2.2, and (**D**) 3.2 ms afterwards.

The overarching goal of our preclinical research was to evaluate the use of in vivo mpUS imaging for classifying normal from steatotic liver. Starting with SWE measurements, Fig. 2 illustrates a temporal sequence of propagating shear waves (0.2–3.2 ms after pulsed US excitation) in control and MCD diet liver. Inspection of these SWE images reveals that shear waves exhibit a slightly lower speed and are more attenuated at distance in MCD diet fed animals at 6 weeks compared to the age-matched controls. From these example cases, the shear wave speed was estimated to be 1.52 and 1.36 m/s for control and MCD diet animals, respectively, whereas the shear wave attenuation was found to be 94.8 and 113.9 Np/m.

**Figure 3:**
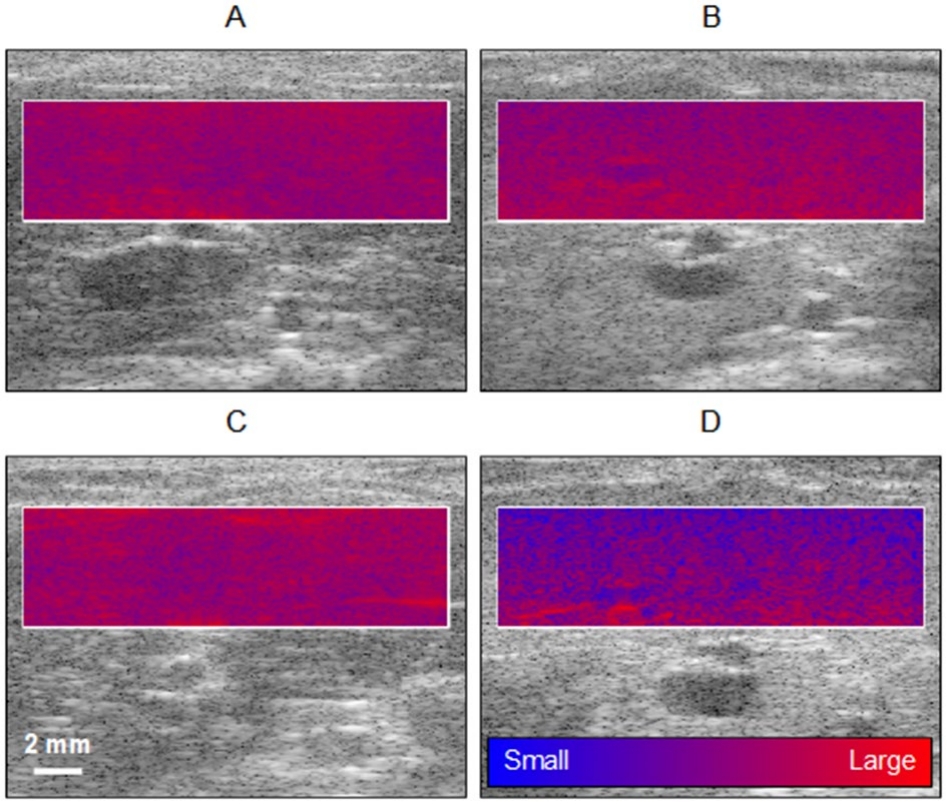
Co-registered B-scan US images with colored H-scan US overlays. Overlapped US images are from (**A**,**C**) control and (**B**,**D**) MCD diet fed animals at (**A**,**B**) 0 (baseline) and (**C**,**D**) 6 weeks. The local H-scan US color display represents (relative) aggregate scatterer size from the liver parenchyma.

B-scan and H-scan US imaging gives insight into backscattered signals from tissue. Representative B-scan US images with an H-scan US colored overlay from the liver parenchyma is presented in Fig. 3. Note the blue color describes local backscattered US signals from smaller sized or less compacted structures and the red color from relatively larger objects. Given the MCD diet fed animals exhibited a notable bluer hue at 6 weeks compared to the time and tissue depth-matched control H-scan US images, this could indicate the presence of macrovesicular steatosis from diffuse fatty infiltration.

**Figure 4:**
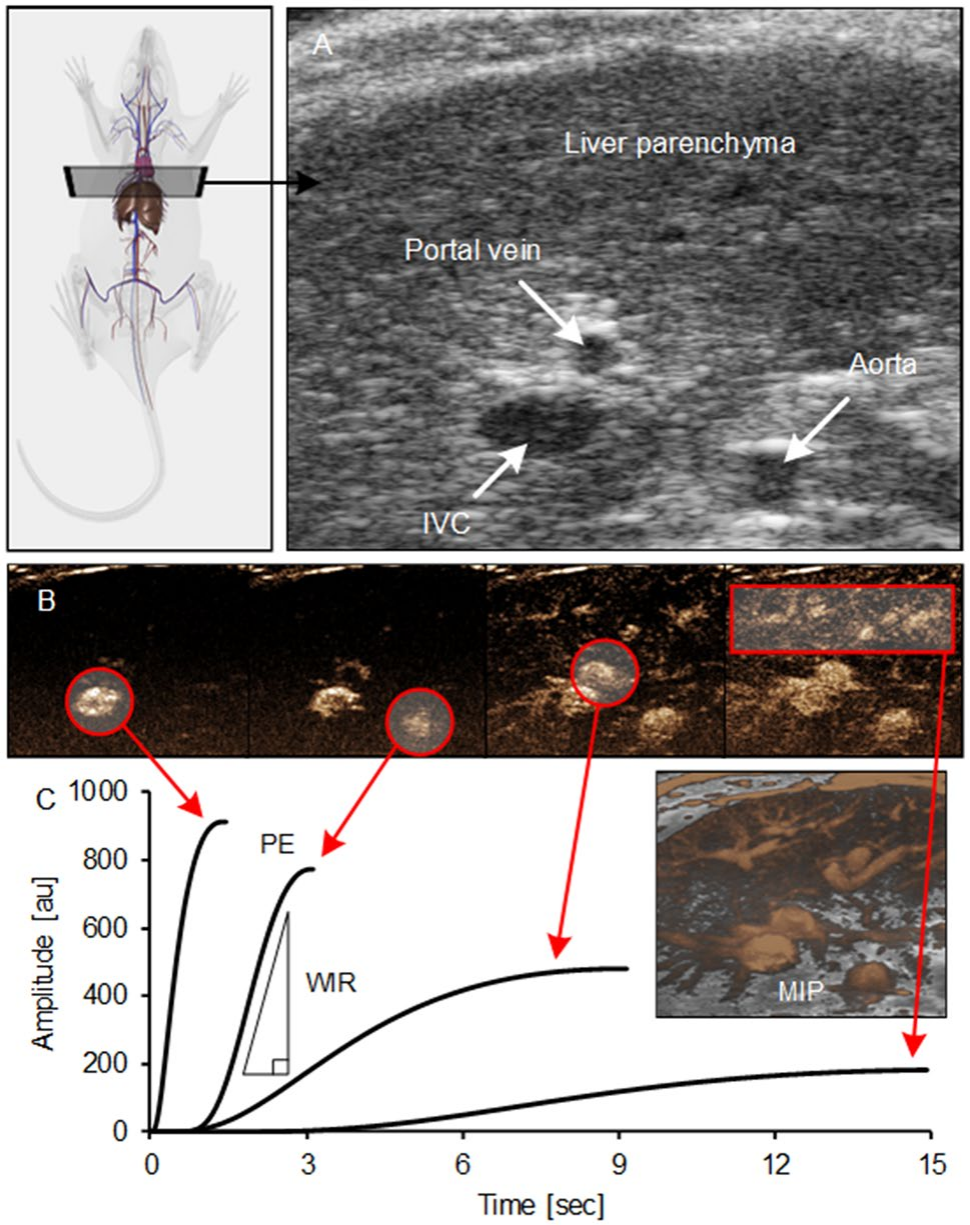
Matched (**A**) B-scan US and (**B**) series of contrast-enhanced US (CEUS) images from a rat liver after administration of a microbubble (MB) contrast agent via tail vein injection. Individual (**C**) time-intensity curves (TICs) describe MB arrival in the major vessels, namely, inferior vena cava (IVC), aorta, and portal vein, in addition to the liver parenchyma from which the peak enhancement (PE) and wash-in rate (WIR) can be calculated. A maximum intentesity projection (MIP) image highlights the spatial distribution of vascular structures. Illustration of basic rat anatomy and indication of the US imaging transverse plane (top left) was generated using commercial software (3D Rat Anatomy v1.30b, Biosphera, São Carlos, Brazil).

CEUS imaging provides insight into liver tissue perfusion and properties of blood flow. To that end, co-registered B-scan US images and a series of CEUS images from control and MCD diet animals were acquired. A typical TIC describes US image intensity values over time in a ROI. As the MB contrast agent was administered via a tail vein catheter, CEUS image enhancement first occured in the IVC, aorta, porta vein, and then the liver parenchyma, Fig. 4. Note that the TIC analysis was limited to the wash-in phase to help minimize recirculation interference after the first pass.

**Figure 5:**
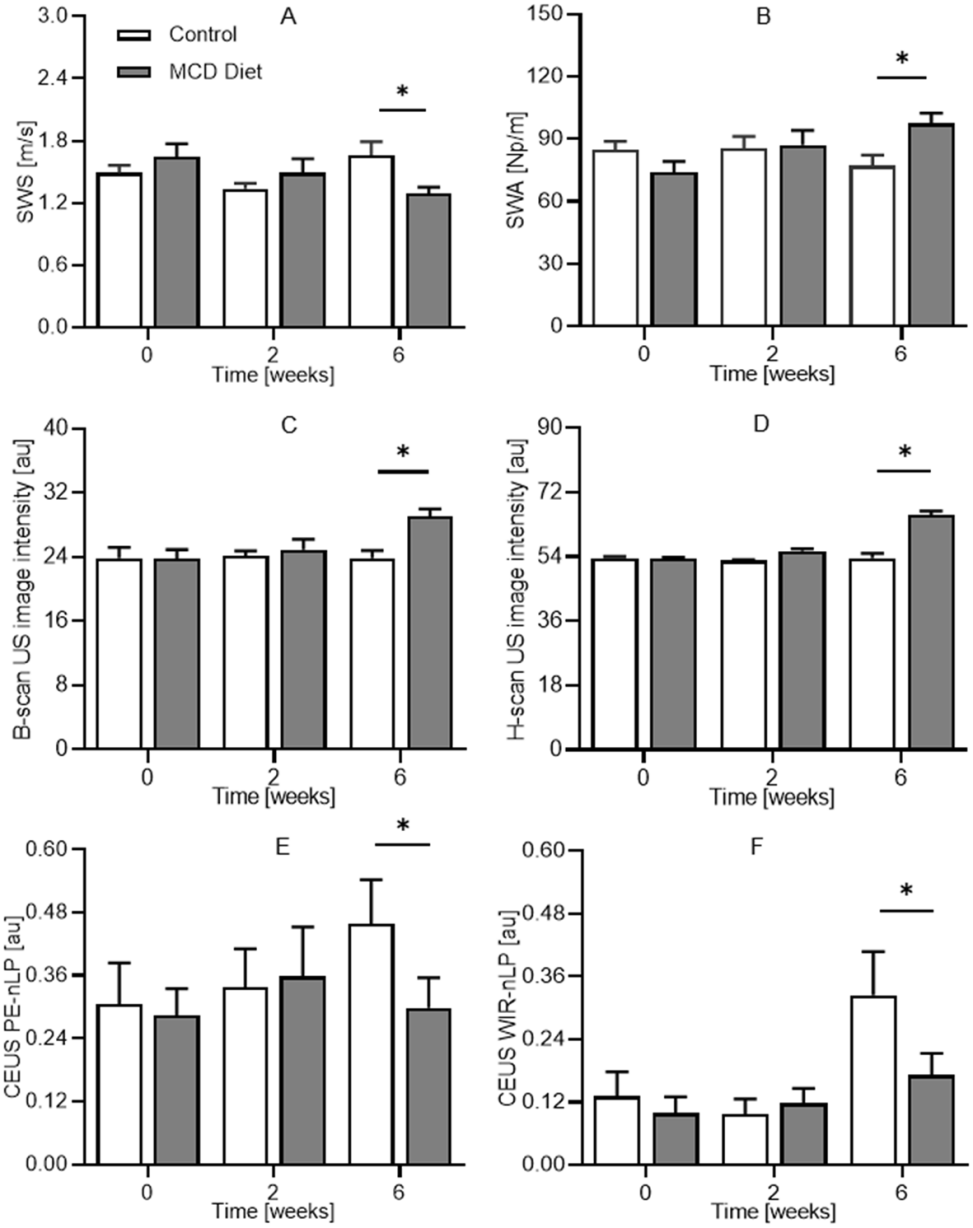
Summary of in vivo mpUS measurements in rat livers at 0 (baseline), 2, and 6 weeks after animals were placed on a control or MCD diet. Boxplots are shown for the following mpUS parameters: (**A**) shear wave speed (SWS), (**B**) shear wave attenuation (SWA), (**C**) B-scan US image intensity, (**D**) H-scan US image intensity, (**E**) CEUS-derived PE, and (**F**) CEUS-derived WIR in the liver parenchyma normalized by co-registered measures from the IVC. A * denotes a *p*-value < 0.05 vs control data.

A summary of all in vivo mpUS image-based group measurements are presented in Fig. 5. Collectively, this data details the progression of mpUS parameters in rat livers at 0 (baseline), 2, and 6 weeks after animals were placed on a standard chow or MCD diet. While no differences were found at baseline (*P* > 0.25) or 2 weeks (*P* > 0.06), all mpUS parameters from the control and MCD diet animal groups at week 6 were found to be significantly different (*P* < 0.05).

**Figure 6:**
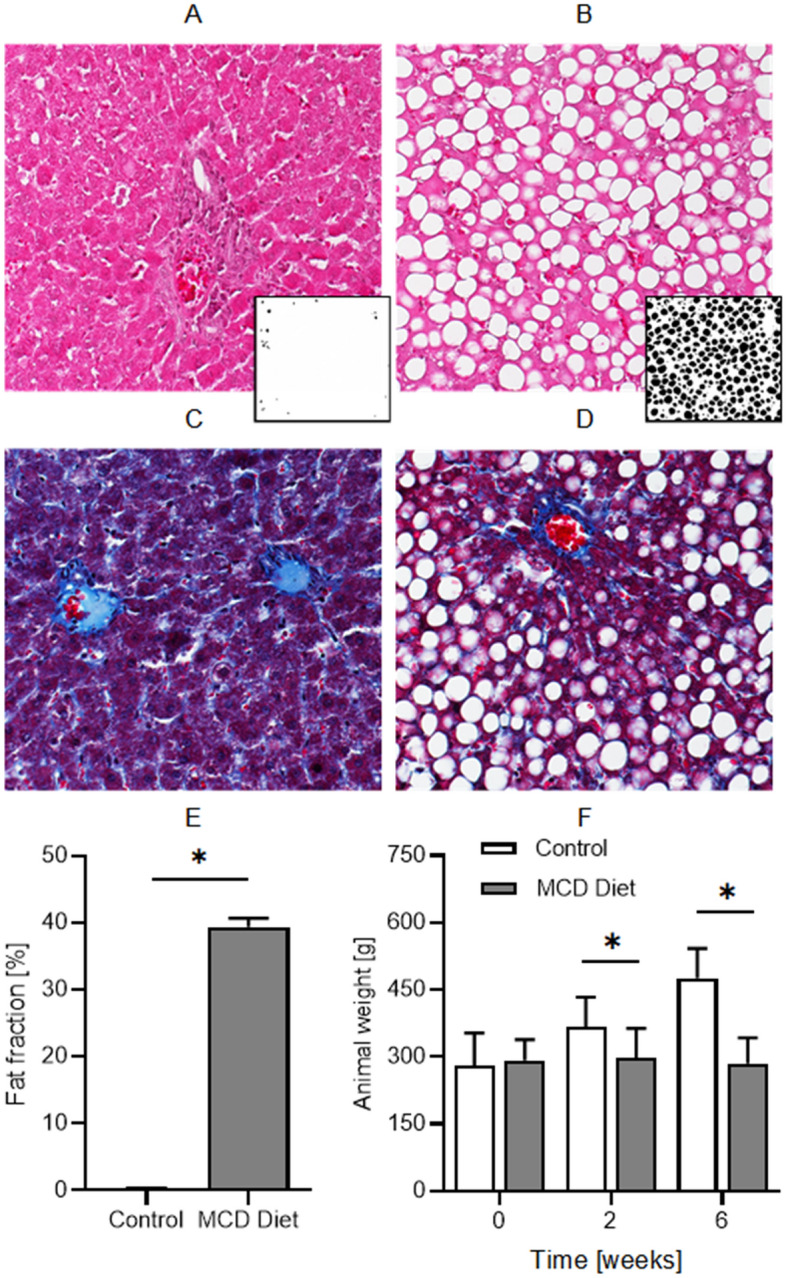
Representative histology images of (**A**,**B**) hematoxylin and eosin (H&E) and (**C**,**D**) Mason’s trichrome stained liver tissue from animals fed (**A**,**C**) control (standard chow) or (**B**,**D**) an MCD diet (bottom) for 6 weeks. Presented are the original H&E histology images and example binarized sub images used to quantify the (**E**) fat fraction score. Note in the H&E the presence of macrovesicular steatosis (white fat deposits) in the MCD diet fed animal livers. (**F**) Summary of tempoeral changes in animal weight.

After the final mpUS imaging session at week 6, all animals were humanely euthanized and livers excised for histological processing. Example liver tissue sections from the control and MCD diet group animals are shown in Fig. 6. These histology images reveal that high grade steatosis was only found in animals fed the MCD diet. This condition is marked by a significant extent of macrovesicular steatosis with little or no inflammatory changes. Instances of focal fibrosis were rare and found with similar likelihood in both animal groups. From binarized H&E images that segmented the diffuse fat deposits, a fat fraction score was calculated. The mean fat fraction from the MCD diet and control group animals was 39.4 ± 1.3% and 0.4 ± 0.1% (*P* < 0.001), respectively. Lastly, while control animals on average got heavier throughout this 6 week study, the animals fed the MCD diet maintained a near constant weight despite the progression of marked steatotic liver disease.

**Figure 7:**
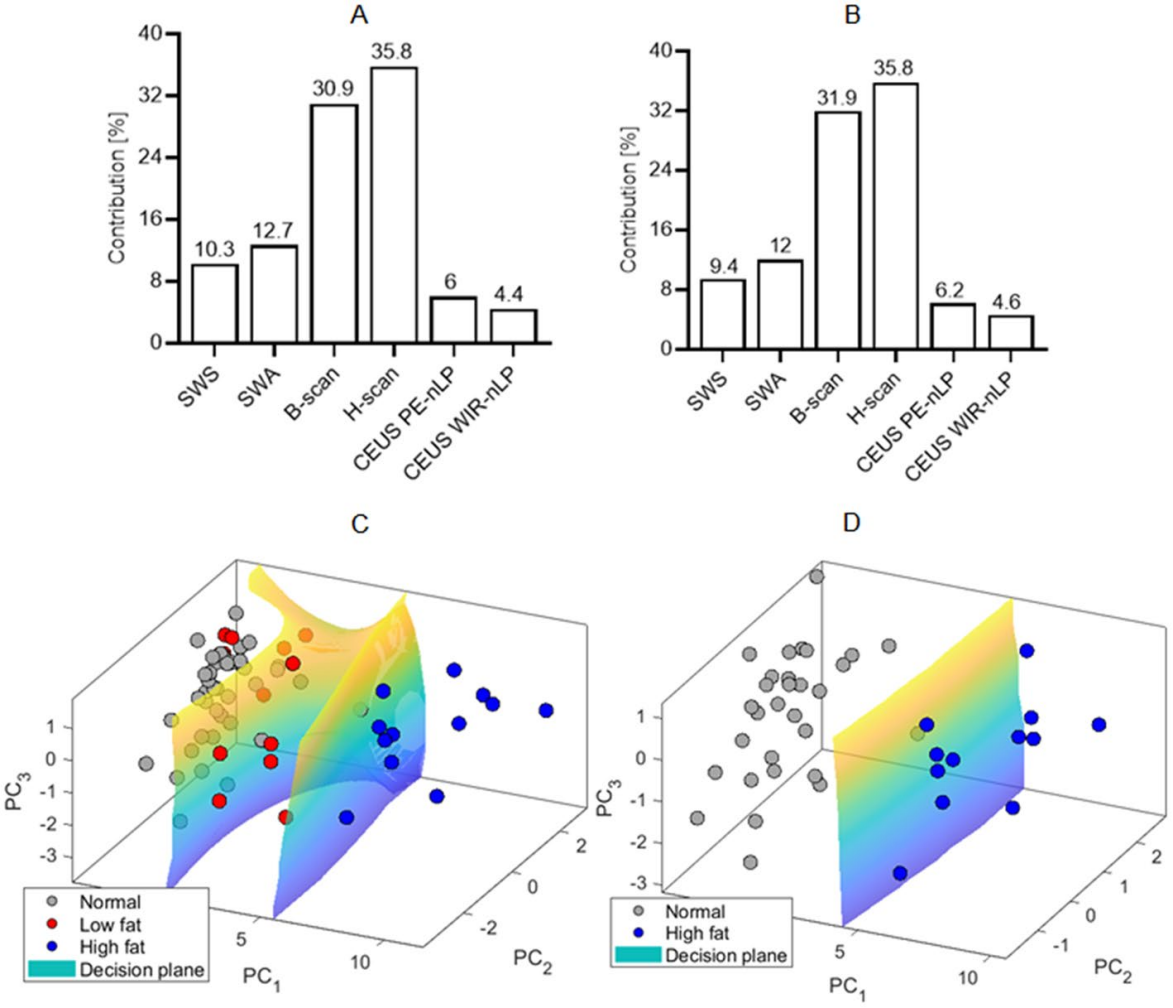
Summary results from principal component analysis (PCA) and support vector machine (SVM) data learning models. Data summarizes the percent contribution from all principal components using PCA for both (**A**) three-category and (**B**) two-category learning. Also shown is the decision plane from the first three principal components using the SVM learning model for (**C**) three-category and (**D**) two-category learning. The low and high fat livers represent the 2 and 6 weeks MCD diet measurements, respectively.

SVM is a supervised machine learning model used to solve classification problems. Herein the mpUS data was used to create an SVM classifier, where mpUS parameters were considered as features in the SVM learning model. Additionally, to visualize hyperplanes in 3D space, PCA reduced the 6 mpUS features into 3 principal components, and its coefficients calculated the contributions from these six features. Figure 7A,B show the contributions of all principal components for three- and two-category learning, respectively. The corresponding decision planes from the SVM classifier are shown in Fig. 7C,D from the first three principal components. In both analyses, H-scan US image measures contributed the highest percentage (> 35%) among the other mpUS data. It is evident from the two-category decision plane that control livers can be separated from high fat livers with near 100% accuracy. The classification accuracy for the three-category decision plane was 89% when only first 3 principal components were used. This accuracy was improved to 92% when all principal components were utilized, which is not shown since 6 features cannot be visualized. To validate the classificiation, when dividing the features into training and testing sets, classification accuracies were 100% and 99%, respectively, for the two-category approach. For the three-category classification, the accuracies were 93% and 82%, respectively.

## Discussion

Quantitative detection of liver fat and fibrosis content is of great importance in the evaluation and staging of NAFLD. The two most prevalent imaging techniques to examine NAFLD patients include magnetic resonance elastography (MRE) and magnetic resonance imaging (MRI)-based proton density fat fraction (PDFF). The former is a technique that builds upon MRI to determine liver stiffness, which is related to the degree of fibrosis. Alternatively, PDFF can accurately determine the amount of fat content in the liver^36^. In a study of 72 patients with biopsy-proven NASH and fibrosis, MRE-based liver stiffness measurements were strongly correlated with fibrosis stage^37^. In this same patient population, MRI-PDFF was shown to have good predictive accuracy for individual grades of steatosis. In a more recent analysis of 370 patients with NAFLD or NASH, PDFF values were also shown to correlate with steatosis grade^38^. Although MRI-PDFF is a useful tool for the noninvasive detection of NAFLD and the quantification of steatosis, MRE is needed to help distinguish between NAFLD and NASH with fibrosis. While promising, one of the major criticisms of MRE and MRI-based PDFF assessments is the equipment and cost associated with MRI scanners, along with the technical expertise required to perform and interpret readings. Hence, MRI may not a suitable candidate for the pharmaceutical industry that aims to develop drugs to treat NAFLD.

Conventional abdominal US is a common imaging modality for subjective evaluation of hepatic steatosis, with good sensitivity and specificity in detecting moderate to severe levels of steatotis (84.8% and 93.6%, respectively^39^. As confirmed by our in vivo data, fat vacuoles within the hepatocytes increase the backscattered US signals, resulting in a brighter (more echogenic) appearance of the liver. Due in part to poor interobserver agreement and the subjective nature of traditional US imaging^40^, more quantitative US-based measurements have been explored. More specifically, several elastography studies have reported sensitivity to identifying NASH with fibrosis in patients with biopsy-proven NAFLD^41–43^. However, recent reports have shown that the sensitivity is improved considerably when SWE information is combined with quantitative US measures of tissue scattering^44^. Franceschini et al. combined spectral-based quantities with SWE to improve classification performance^45^. It was also been shown that the combination of three US parameters (stiffness, effective scatterer size, and acoustic concentration) provides the best classification performance when compared to classifications obtained from the spectral-quantitative US or stiffness parameters alone. However, this study was conducted on ex vivo liver samples and did not demonstrate the ability to distinguish NASH from fibrotic tissue. In another study, it was demonstrated that the classification of NASH can be improved when SWE is combined with quantitative US parameters^44^. The area under the receiver-operating characteristic curve (AUROC) increased from 0.63 for SWE alone to 0.72 for a model that combined elastographic and quantitative US techniques.

Herein we introduced an in vivo mpUS imaging approach that incorporated shear wave parameters to determine viscoelasticity, CEUS to evaluate liver vascularity and perfusion, and H-scan US to estimate tissue microstructural information. The formation of steatosis leads to a decrease in liver tissue shear wave speed measures and an increase in attenuation^32,46^. However, the shear wave speed also increases with increasing stiffness, where this can be observed from control measures where the liver appeared to stiffen with age^47,48^. H-scan US imaging was used to obtain the microstructure properties of liver tissue by analyzing backscattered US signals. Progressive accumulation of fatty deposits in the liver altered the spectral content, leading to a significant blue shift in the H-scan US image intensity in the MCD diet animals. With increasing liver fat content, it has been shown that the effective scatterer diameter and density tends to decrease^49^. Lastly, CEUS imaging is sensitive to fatty liver disease progression and can accurately detect vascular features of NASH^20^. It was shown that the CEUS-based PE and WIR parameters decreased in fatty livers and consistent with previous studies^20,23^. Fat accumulation in the liver parenchyma can produce microvascular constriction and blood flow resistance during the pathologic progression of NAFLD disease. As the liver is up to 30% blood by volume^50^, the longitudinal increases in PE and parameters in the control livers were presumably due in part to natural organ growth. This assumes that larger blood vessels contributed to a higher blood volume and lower vascular resistance. These physical changes in vascular size may have also contributed to the observed changes in the H-scan US images^51^.

Our results demonstrated that mpUS measurements between control and MCD diet fed animal groups were all significantly different at 6 weeks (*P* < 0.05). Consequently, the decision plane from the SVM classifier efficiently separated the data clusters, leading to a 100% classification accuracy after two-category training. We processed the features with Z-score normalization and PCA, to find the individual mpUS parameter contributions. The PCA results showed that the H-scan US image features contributed the most among the six mpUS measurements evaluated. This was attributed to the large proportion of fat deposits that accumulated in the liver, which was confirmed by histology images and the fat fraction score. However, the classification accuracy was reduced to 92% in three-category training, where it classified normal, low fat (2 weeks), and high fat (6 weeks) livers. In this case, the mpUS measurements fail to reach the highest accuracy as there is overlap between the normal and low fat livers. Further studies will include advanced processing algorithms to improve the H-scan US and SWE parametric estimations. Repeat CEUS studies will also help minimize measurement variance. Collectively, these advances should help improve the classification accuracy and detection of early stage NASH. Also, experiments will be repeated and additional histology will be performed at 2 weeks for comparison to the in vivo mpUS findings. This is a noted limitation of the current study. This confirmation might provide more insight into the NAFLD spectrum and the effectiveness of mpUS parameters at different stages of liver disease progression. Further, we will examine the mpUS data classification accuracy with a more detailed comparison to time-matched histology samples. This work may also necessitate use of a different animal model that more closely recapitulates human disease and exhibits the spectrum of NAFLD ranging from simple steatosis through NASH and late-stage fibrosis.

The proposed mpUS image-derived biomarkers of liver tissue appear to be a promising approach for the early assessment of NAFLD. This study showed the effectiveness of mpUS imaging measurements for monitoring the progression of steatosis, thereby introducing the potential for the early detection of NASH. Overall, we envision that noninvasive mpUS imaging could evolve to be used as a surrogate biomarker for the current clinical standard of the invasive liver biopsy.

## Methods

### Animal model of NAFLD

All procedures were carried out in accordance with relevant guidelines and regulations. Animal experiments were performed based on a protocol approved by the Institutional Animal Care and Use Committee (IACUC) at the University of Texas at Dallas. This manuscript complies with the ARRIVE guidelines for reporting animal research^52^. The MCD diet is a classic dietary model for studying NAFLD. This diet is high in sucrose and fat and is deficient in methionine and choline, which results in increased fat accumulation in the liver. A population of 12-week-old Sprague–Dawley rats (*N* = 21; Charles River Laboratories, Wilmington, MA) were randomly divided into two groups, namely, control (*N* = 9) and MCD (*N* = 12). Control animals were fed standard chow, whereas the MCD group received a special diet (MP Biomedicals, Solon, OH). Animals were kept under a 12-h day/night rhythm with free access to food and water.

### Liver imaging protocol

Animals were anesthetized with 1–2% isoflurane in oxygen (V3000PK, Parkland Scientific, Coral Springs, FL) and placed on a temperature-controlled heating pad to maintain core levels (Rodent Surgical Monitor, AnimaLab, Poznan, Poland). Prior to mpUS imaging, a catheter was placed and secured in the tail vein as was needed for MB dosing during CEUS imaging. To acquire the sequence of mpUS measurements, CEUS and H-scan US scans were performed by a Vevo 3100 system (FUJIFILM VisualSonics Inc, Toronto, Canada) equipped with an MX201 linear array transducer, and SWE was performed using a Vantage 256 US system (Verasonics Inc, Kirkland, WA) equipped with an L11-4v probe. For each animal, the US transducer was positioned and fixed in the transverse plane after co-visualization of major blood vessels, including the aorta, inferior vena cava (IVC), and portal vein. Identifying and imaging at the level of these anatomical landmarks helps in the acquisition of information from similar planes, particular during the repeat mpUS imaging sessions.

A custom SWE imaging sequence was implemented on the programmable Vantage 256 US system. The focused US push pulse frequency (5.2 MHz) were set within the lower -6 dB of the transducer bandwidth to broaden the push beam while maintaining high transmit power efficiency^53^. The push beam used an aperture size of 64 elements. Our sequencing involved three focused US pulses at varying levels of tissue depth (2 mm spacing between pulses, pulse length of 1200 cycles or 230 µs) to create a series of propagating shear waves that interfere constructively to create two intense shear waves^54^. The use of ultrafast plane wave US imaging permitted displacement tracking of these shear wave propagation patterns. A 2-dimensional (2D) algorithm estimated tissue displacements from the beamformed in-phase quadrature (IQ) data^55^. Thereafter, a 2D Fourier transform was applied on the tissue displacements to estimate the shear wave speed and attenuation parameters^56^. During SWE, the US push pulse focuses were positioned in the bulk liver parenchyma and a collection of four measurements (repeated 10 times) per subject were taken at slightly different locations.

H-scan US images were reconstructed from radiofrequency (RF) data collected using the Vevo 3100 scanner (center frequency = 15 MHz). The RF data were corrected for signal attenuation using a global scaling value of 0.3 dB/cm/MHz. Two parallel convolution filters were applied to the raw data sequences to measure the relative strength of the received signals relative to *GH*_2_ and *GH*_8_. Signals were then normalized by the signal energy $\sqrt {E_{n} }$ before bandpass filtering (5–18 MHz), and calculation of the signal envelope using a Hilbert transform. The relative strength of these filter outputs were color coded whereby the lower frequency (*GH*_2_) backscattered US signals were assigned to a red (R) channel and the higher frequency (*GH*_8_) components to a blue (B) channel. The envelope of the original unfiltered data was used to generate the B-scan US image. H-scan US image reconstructions were performed on at least 100 frames of data and region-of-interest (ROI) analysis yielded a mean measurement of relative scatterer size^26^. H-scan US image intensity was calculated as a ratio of the B channel to the sum of the R and B channel components.

Lastly, a controlled bolus injection of a MB contrast agent (Definity, Lantheus Medical Imaging) was slowly administered (50 µL over a 10 s period using a syringe pump). CEUS images were then collected using the Vevo 3100 system with a nonlinear contrast imaging mode (center frequency = 12.5 MHz). Imaging was performed immediately before and after MB injection for at least 30 s to capture blood perfusion kinetics throughout the liver cross-section. Note that CEUS occurred last as the presence of any residual MBs in circulation can induce considerable bioeffects when exposed to SWE and high-intensity US. The temporal sequence of CEUS images were processed using commercial software (Vevo Lab v3.2.5, FUJIFILM VisualSonics, Inc). After ROI placement around the IVC and liver parenchyma, a time-intensity curve (TIC) for each was generated after model fitting and used to extract the peak enhancement (PE) and wash-in rate (WIR) parameters, which are surrogate measures of blood volume and flow rate, respectively. PE and WIR estimates from the liver parenchyma were normalized by the measures from the IVC to obtain normalized PE (PE-nLP) and WIR (WIR-nLP) parameters. Additionally, TIC data was exported as a delimited text format and plotted using Excel (Microsoft Office Professional Plus 2019, Microsoft Corp, Redmond, WA) and Visio (Microsoft Visio Professional 2019, Microsoft Corp) software for display purposes only.

### Data classification

Using MATLAB R2020b (MathWorks Inc, Natick, MA), we analyzed six mpUS measurements using PCA and SVM to predict fatty liver conditions. Since each mpUS measurement has a different scale that causes a different weight, a Z-score normalization was performed prior to classification. The normalized mpUS measurements were used by an SVM model that uses a Gaussian kernel function to construct non-linear hyperplanes. The model was trained and tested using 80% and 20% of the dataset, respectively. More details for implementing the classifier and optimizing the SVM parameters can be found in^57^. Here, we trained the SVM classifier with two different input datasets. First, learning (three-category) included all measurements at 0, 2, and 6 weeks to classify normal, low fat, and high fat livers, whereas the other learning (two-category) only included measures at 0 and 6 weeks to classify normal and high fat livers. Herein, the normal group refers to measurements at 0 week and control at 2 and 6 weeks. The low and high fat livers refer to MCD diet measurements from 2 and 6 weeks, respectively. In each training, the SVM model constructed decision planes to differentiate the liver conditions. For visualization of decision planes, we used PCA to reduce the number of parameters, and then the first three prinicpal components were assigned as SVM inputs.

### Histological processing of liver tissue

After the last mpUS imaging session at 6 weeks, animals were euthanized and livers excised. The right medial and left lateral lobes of the liver (≥ 50% of each lobe) were fixed in 10% neutral-buffered formalin for at least 7 days at room temperature. Liver tissue was then embedded in paraffin, sectioned (5 μm), and mounted. Hematoxylin and eosin (H&E) stains were used for morphological analyses, and Masson's trichrome stain (Sigma-Aldrich, St Louis, MO) for assessment of hepatic fibrosis. Digital images were acquired with a microscope at a magnification of 40–100 × (Axio Scope.A1, Carl Zeiss, Thornwood, NY). H&E images were binarized and processed with morphological operations before quantification of the percent fat fraction.

### Statistical analysis

Parametric group measurements were summarized as mean ± SD. Longitudinal changes were assessed using a repeated measures analysis of variance (ANOVA) test. A comparison of control and MCD diet group parameters were evaluated using a Mann–Whitney *U* test. A *P*-value less than 0.05 was considered statistically significant. All statistical analyses were performed using Prism 9 (GraphPad Software, San Diego, CA).
